# A Scoping Review of Three Dimensions for Long-Term COVID-19 Vaccination Models: Hybrid Immunity, Individual Drivers of Vaccinal Choice, and Human Errors

**DOI:** 10.3390/vaccines10101716

**Published:** 2022-10-14

**Authors:** Jack T. Beerman, Gwendal G. Beaumont, Philippe J. Giabbanelli

**Affiliations:** 1 Department of Computer Science & Software Engineering, Miami University, Oxford, OH 45056, USA; 2 IMT Mines Ales, 6 Av. de Clavieres, 30100 Ales, France

**Keywords:** agent-based model, COVID-19, SARS-CoV-2, vaccine acceptance, variants

## Abstract

The virus that causes COVID-19 changes over time, occasionally leading to Variants of Interest (VOIs) and Variants of Concern (VOCs) that can behave differently with respect to detection kits, treatments, or vaccines. For instance, two vaccination doses were 61% effective against the BA.1 predominant variant, but only 24% effective when BA.2 became predominant. While doses still confer protection against severe disease outcomes, the BA.5 variant demonstrates the possibility that individuals who have received a few doses built for previous variants can still be infected with newer variants. As previous vaccines become less effective, new ones will be released to target specific variants and the whole process of vaccinating the population will restart. While previous models have detailed logistical aspects and disease progression, there are three additional key elements to model COVID-19 vaccination coverage in the long term. First, the willingness of the population to participate in regular vaccination campaigns is essential for long-term effective COVID-19 vaccination coverage. Previous research has shown that several categories of variables drive vaccination status: sociodemographic, health-related, psychological, and information-related constructs. However, the inclusion of these categories in future models raises questions about the identification of specific factors (e.g., which sociodemographic aspects?) and their operationalization (e.g., how to initialize agents with a plausible combination of factors?). While previous models separately accounted for natural- and vaccine-induced immunity, the reality is that a significant fraction of individuals will be both vaccinated and infected over the coming years. Modeling the decay in immunity with respect to new VOCs will thus need to account for hybrid immunity. Finally, models rarely assume that individuals make mistakes, even though this over-reliance on perfectly rational individuals can miss essential dynamics. Using the U.S. as a guiding example, our scoping review summarizes these aspects (vaccinal choice, immunity, and errors) through ten recommendations to support the modeling community in developing long-term COVID-19 vaccination models.

## 1. Introduction

Most Americans have come to agree that “we will never be rid of COVID-19 in our lifetime” [1]. Although vaccines and the ongoing development of antiviral drugs are great achievements [2,3], COVID-19 has continued to wreak havoc in several ways. During the first half of 2022, more than 170,000 individuals died from COVID-19 in the U.S., amounting to over a million deaths since the emergence of the virus [4]. In addition, COVID-19 continues to exert a significant toll on the economy through disruptions in the workforce. As COVID-19 has exacerbated existing systemic challenges, the temporary matter of sickness-related absences in the early days of COVID-19 [5] is now amplified by broader issues manifested through waves of resignation. For example, many nurses or teachers are leaving the profession [6,7], with departures being even more pronounced in places that already experienced staffing shortages before COVID-19 [7]. This phenomenon, “The Great Resignation”, is not limited to healthcare or academia, and is the theme of dozens of articles that document labor shortage across occupations [8,9,10]. As a result, several efforts are underway to transition to a post-pandemic future, such as shifting work arrangements with a growing part of remote work [11].

Despite our logistical preparedness, there are reasons to be concerned when we account for two phenomena. First, and most importantly, the effectiveness of our vaccines is decreasing against new strains, which leads to (re)infections. Indeed, many Americans have already had COVID-19 multiple times [1] and the spread of immune-evasive subvariants is fueling the growth of reinfections. For instance, California went from 1 in 20 reinfections cases at the beginning of 2022 to 1 in 7 by July [12]. Currently available COVID-19 vaccines were developed for a prototype strain, but variants such as Omicron have more than 30 mutations in the spike protein of the prototype strain, which is essential for infection as it initiates the host cell entry [13]. Consequently, “available FDA-approved and -authorized COVID-19 vaccines are less effective against currently circulating virus variants than against previously circulating strains of virus” [14]. This effect is demonstrated since most infections in the U.S. as of June 2022 are attributed to two Omicron sublineages (BA.4 and BA.5) [14]. Second, the problem is compounded by our shifting responses and attitudes to the pandemic. A survey in June 2022 reported that half of U.S. adults wear a face mask “when away from home, the lowest since the start of the pandemic” [15]. Another survey showed that the proportion of people bothered when others do not mask in public fell to an all-time low, at 18% among Republicans and 52% among Democrats [16]. On the one hand, we see a diminishing use of face masks, lower vaccine effectiveness, and a lower intended receipt of COVID boosters [17]. On the other hand, we likely need annual vaccine composition updates [18]. Surveys in Jordan show that less than 1 in 5 persons would take an annual booster [19], while about 2 in 3 Canadians [20] and more than 4 in 5 German-speaking adults would be willing [21]. Results in the U.S. are mixed, as surveys were conducted across sub-populations with different profiles [22].

The Modeling & Simulation (M&S) approach of *Agent-Based Modeling* (ABM) has been used to model COVID-19 since the second half of 2020, because of the approach’s ability to track individual agents who can have different attributes, different decision-making processes, and be exposed to different environments [23]. This ability to implement heterogeneity is indeed essential for COVID-19 since it is found in individual risk factors (e.g., age, hypertension, diabetes), contact patterns (e.g., social networks in the community or work settings), behaviors (e.g., willingness to be vaccinated), and spatial aspects (e.g., access to healthcare) [24,25,26]. Although ABMs for COVID-19 have been extremely varied in purpose and design [27], their design broadly followed three stages in the pandemic [28]: ABMs started with a handful of stages (e.g., susceptible, exposed, infected, recovered) and examined which non-pharmaceutical interventions would have the strongest effect [29,30,31], then ABMs were created to support vaccine-related studies (e.g., who should be vaccinated, where to place the centers and how to staff them) [32,33,34] and lastly, the current wave of studies where repeated boosters account for waning immunity [35,36]. 

Truszkowska and colleagues provided one of the first models in which immunity was totally and gradually lost over the course of six months [35]. This approach included vaccine hesitancy, which was captured by assuming that only a percentage of the population would get vaccinated [35]. More recently, Kelly et al. proposed a comprehensive model that accounts for the waning vaccine induced immunity and naturally acquired immunity, as well as seasonal patterns in infection, and the emergence of annual or biannual variants [36]. Again, vaccine hesitancy was modeled population-wide by assuming either a high booster intake (85% to 98%) of individuals 50 years of age or higher with comorbidities, or modeled with a reduced intake of 50%. These models concluded that a high adherence to frequent booster doses is necessary to avoid future outbreaks and prevent a burden on the healthcare system—a conclusion shared by studies using differential equations [37,38]. Since adherence to frequent boosters is the key but it was only modeled by assuming a percentage across wide sub-populations, it is important for future ABMs to address this research gap by detailing how individuals decide to get vaccinated as a function of sociodemographic, health, psychological, and information-related variables. 

Several open-source COVID-19 ABMs can be reused and extended by the research community, including COVASIM [39], OpenABM-COVID19 [40], and COMOKIT [41]. These packages and other individual-based epidemiology simulation kits [42,43] allow us to create a virtual population that reflects several demographic indicators (e.g., age, sex) and their impact on disease spread (e.g., age-linked disease progression and mortality). Packages such as COVASIM also embed each individual in several networks (home, community, work, school) based on age (e.g., children go to school, adults to work). Through revisions, the current version (3.1.2 from 16 January 2022) also captures “co-circulating variants, imperfect immune memory, and multiple vaccines” [44], tracks the properties of variants (relative transmissibility compared to wild type, relative severity, relative immunity, variant-specific protection of vaccines), and allows users to add custom variants. The popularity and reuse of such packages is demonstrated by their applications to multiple settings such as Australia [45,46,47], the UK and/or US [48,49,50], Vietnam [51], or Italy [52], at both the country- and city-scale [53]. Our scoping review thus emphasizes elements that are commonly missing across these packages in order to have a broad representation of the current technical capabilities in the field.

The *main contribution of our scoping review is to support the development of the next generation of ABMs* by examining three aspects: vaccine hesitancy, joint occurrence of natural- and vaccine-induced immunity, and the inclusion of human errors in decision-making. Specifically, we synthesize the current evidence base and identify research gaps, leading to 10 recommendations on aspects to include in future models, alongside their operationalization. The recommendations are featured prominently (as **R1**, **R2**, etc.) throughout the sections.

The review is organized as follows. Section 2 starts with the concept of immunity and its representation in models. Then, Section 3 summarizes the evidence base regarding strong drivers of vaccinal choice. We cover the last dimension in Section 4 by explaining how individuals are not perfect and can engage in several types of errors during their decision-making processes. The last two aspects have received less attention in modeling packages, hence these two sections include detailed suggestions for inclusion in future models. Finally, Section 4 discusses a list of limitations inherent to the modeling process and limited current knowledge on variants and individual choices.

## 2. Immunity: Variants, Waning Effect, and Hybrid Cases

Early models of COVID-19 that included vaccines were developed to help plan ahead. For instance, models would determine the most effective locations to setup vaccination centers or prioritize certain sub-populations that should be first administered vaccinations. In the simulated timespans of these models, it was adequate to consider that individuals would have a *constant* level of immunity once recovered or vaccinated, and that the dominant strain at the time was the *only one*. As we transition into a more long-term perspective, we need to account for changes in the level of immunity, including the cumulative effect of vaccination and recovery (particularly since most Americans have had COVID-19 [54]), as well as the ongoing emergence of variants.

As shown in Table 1, recent models have handled immunity differently. Most models use a gradual decay, with arguments in favor of a Gamma distribution rather than an exponential one (to reflect that the initial immunity remains at a high level for several weeks instead of declining immediately). This is represented either by the direct use of a fitted Gamma distribution, or by the ‘linear chain trick’ which consists of use a sequence of exponentially distributed decays (e.g., agents go through stages V_1_, …, V_5_ back-to-back) [55,56]. 

The models either did not account for the synergistic effect of natural infections *together with* vaccinations, or created a dedicated stage without specifying the corresponding transitions. For example, a model considered that a vaccinated person who became infected *lost* the vaccine-induced immunity and only gained natural immunity upon recovery, hence making the two forms of immunity mutually exclusive [58]. However, the hybrid case resulting from natural infection and vaccination confers the most robust and durable immunity [60,61,62]. Studies on immunology on different variants and various countries have found that hybrid immunity was protective against reinfection and severe disease outcomes [63,64,65,66]. As summarized by Bates and colleagues, “the additional antigen exposure from natural infection substantially boosts the quantity, quality, and breadth of humoral immune response regardless of whether it occurs before or after vaccination” [67]. Newer models are starting to include this effect, for example by assuming that a prior infection counts as a single vaccination, hence the ‘actual’ vaccination has the effect of a booster [68]. A recent retrospective cohort study in Sweden [69] confirmed several of the numbers in the OpenCOVID model and sheds light on the effect of the synergistic effect of hybrid immunity. The study showed that natural immunity conferred a 95% lower risk of infection and 87% lower risk of hospitalization for up to 620 days, which closely aligns with OpenCOVID. The study further established that hybrid immunity provided an additional 58% (for one dose) or 66% (for two doses) reduction in infection compared to natural immunity, and lasted up to 9 months with mixed findings on attenuation. Although numerous knowledge gaps remain [70], findings are starting to emerge regarding mediating factors in the immune response created by receiving a vaccine after a natural infection. In particular, a study using the U.S. Military Health System further suggested that the timing between a prior infection and vaccination was highly predictive of immune response, while prior disease severity did not play a role [62]. 

The prevalence of hybrid immunity is rising quickly; a cross-sectional study of blood donors in the U.S. found an increase from 0.7% of blood samples with hybrid immunity in January 2021 to 18.9% by December [71]. A review by Bhiman and Moore listed several scenarios as potential consequences of this ongoing increase in the hybrid immune population [72]; the inclusion of such scenarios in future computational models may improve our ability at forecasting population-wide trends.

Few models examined the continuous emergence of new variants. For example, in the OpenCOVID model [36], each new variant retained the same severity and was assumed to be 25% more infective as well as 25% more able to evade immunity than its predecessor. Another model considered that new variants would be 3.5-fold as virulent compared to the wild-type [55]. Although models assumed a *regular* emergence of new variants, illustrative examples [73] explain that the emergence depends on the number of circulating cases (I) and susceptible individuals (S) as well as the rate of infection (β) and the chance of each infection to create an escape variant by mutation (μ). Thus, the time until the first escape mutation appears is approximated as an *exponential* of mean

(1) $$\frac{1}{SI\beta \mu }\;$$

Based on sensitivity analyses on the models, scholars determined that the primary effect of vaccines was to block infections (80%) and secondarily of avoiding severe disease outcomes (the remaining 5%) [36]. Simulation results from COVASIM [55] on vaccine design further suggest that, going forward, creating a broadly neutralizing vaccine would be more beneficial (40% reduction in death) than a more durable vaccine (10% reduction in death). However, the effect would be highly reliant on releasing the vaccine at the right time, hence the preferred solution would be a vaccine that is both durable and broadly neutralizing (65% reduction).

Given the evidence base, we recommend that future models include:

**(R1)** a *gradual decay in immunity* by using a Gamma distribution, for instance through the creation of intermediate states (i.e., the ‘linear chain trick’).

**(R2)** *hybrid immunity*, which confers the most robust and durable immunity; this may be approximated by counting a prior infection as having the effect of a booster dose.

**(R3)** the *ongoing emergence* new variants that partially evade immunity, with a timing depending on circulating cases rather than at fixed time intervals. 

## 3. Vaccinal Choice

### 3.1. Drivers of Vaccinal Choice

Numerous studies have been conducted to identify socio-demographic factors related to an increased or decreased willingness to take vaccines. Using the U.S. population as a guiding example, sample studies relevant to this context are listed in Table 2. Note that models of COVID-19 have not yet accounted for the many drivers of individual vaccination choices, instead opting to either use a population-wide percentage of vaccinated individuals [35] or account for age [33,52]. This occurred even in highly configurable frameworks where agents execute detailed plans based on their health, demographic, and organizational roles [74]. Two models accounted for the level of caution (rising with case numbers), sense of safety (rising with vaccination), or perceived vaccination risk, but at the population-level rather than via individuals [75,76]. Given Table 2, we recommend that future models include:

**(R4)***multiple socio-demographic factors* (e.g., age, sex, race and ethnicity, annual income, college degree, key comorbidities, political party) that were found to strongly predict vaccinal choices. 

### 3.2. Capturing Drivers in a Model: The Role of Data and Sequential Agent Initialization

**Figure 1:**
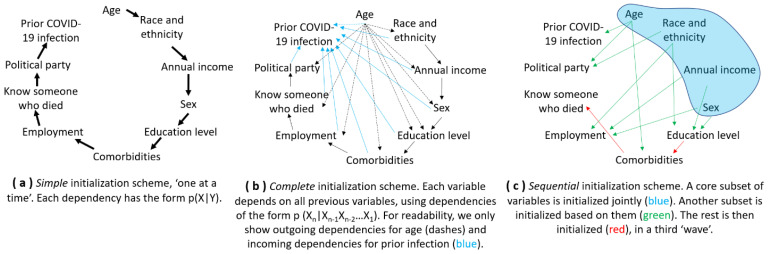
A simple initialization scheme (**a**) would ignore several important dependencies in the data, but capturing a large number of dependencies (**b**) may be infeasible given data limitations. A practical approach is thus to initialize the agents’ attributes in consecutive waves (**c**).

When agents possess multiple traits, the values may not be initialized *independently*, one at a time (Figure 1a). Indeed, values can be *correlated*: for instance, comorbidities tend to raise with age. Multiple correlations can exist, as is the case with the dependency of annual income both on employment and on educational attainments. Considering that every factor may depend on all other factors would be infeasible, as it leads to circular dependencies. Even a scheme where each factor depends on all those previously initialized can be practically infeasible, since it would be arduous to find a nationally representative dataset that covers all factors (Figure 1b). Consequently, an initialization scheme will have to retain *some* of the dependencies based on the data available [83] (Figure 1c). In this subsection, we illustrate how these considerations can be addressed by using U.S. data.

Consider that we aim to create a virtual population of agents with multiple target socio-demographic factors. To start, we identify a data source that contains as many as possible of the important attributes *together*. In our case, the US Census Current Population Survey (CPS) 2019 [84] can serve to create a pool of agents who are *jointly* assigned an age, income, race and ethnicity, and sex. That is, all four attributes can be assigned simultaneously, thus capturing their interdependencies in the population. Then, additional data sources will need to be found to cover the remaining aspects. Note that these sources need to also include some of the previously initialized attributes, otherwise dependencies cannot be adequately reflected. For example, if a dataset contains the prevalence of prior COVID-19 infection based only on data per county, then there would be no way to link the data to individuals characterized by age, race and ethnicity, annual income, or sex. In the case of the U.S., several data sources can be used as shown in Table 3. 

Finally, there can be a gap between the factors used in studies on social determinants of health and the content of national surveillance systems. For example, studies reported that comorbidities were a strong determinant of vaccinal choice (Table 2), but different surveillance systems may track different types of *specific* comorbidities, hence there will be a need to translate a high-level concept into specific conditions and work across additional sources. A pitfall for modelers is to misunderstand the notion of comorbidities. Since comorbidities refer to other diseases present in a patient, death reporting systems may show pneumonia as a prevalent comorbidity, hence modelers could (erroneously) use pneumonia as an agent’s attribute for comorbidity. However, pneumonia is a *complication* of COVID-19, rather than an underlying medical condition that increases the *risk* profile of a person if they get infected. It is thus important to carefully translate high-level constructs into specific ones given the context. In this example, comorbidities as risk factors may include hypertension and diabetes since they are commonly found in patients hospitalized with COVID-19 worldwide [85,86], while noting that the evidence-base is weaker for hypertension once we control for other risk factors such as age [87]. Dependencies matter across comorbidities as well, since diabetic agents have an elevated risk for hypertension; Table 3 thus initializes diabetes and then hypertension.

In sum, a satisfactory initialization of several attributes in the agents will require:

**(R5)** the identification of several data sources that share factors to allow *data linkages*.

**(R6)** an initialization scheme that satisfies *multiple core dependencies* between factors by initializing them in a specific batch order.

**(R7)** a careful *contextual translation* from high-level constructs onto specific variables offered in different surveillance systems and reports.

**Table 3:** Creation of a large pool of agents by first initializing four attributes together, then determining the remaining five by accounting for several interdependencies.

			1st Wave of Initialization of Four Categorical Features Jointly [80]			
	Ref	Factors	Age	Income	Race and Ethnicity	Sex
**2nd wave of initialization**	[88,89]	Bachelor’s degree	✓		✓	✓
	[90,91]	Political party ^1^			✓	✓
	[92]	Diabetes	✓		✓	✓
	[93,94]	Hypertension ^2^	✓			✓
	[95]	≥1 dose of vaccine	✓		✓	

^1^ Two steps are involved: first we apply a population-wide probability of voting (agents who do not vote are ‘undecided’), then we subdivide agents into Democrats and Republicans based on sex, race and ethnicity. ^2^ Hypertension is created after agents have been assigned a level of diabetes, to ensure that diabetic agents have twice the risk for hypertension [93].

Note that a model is necessarily a simplification and *data availability may govern some of these simplifications*. For example, a modeling team may not have access to data for some of the strong drivers of vaccinal choice (Table 2), hence agents may not possess the corresponding attributes. For instance, sociodemographic information may not include occupations, hence a model may lack the notion that certain professions are at higher risk (e.g., medical professionals) or face specific obligations (e.g., annual vaccine). 

### 3.3. Extending an Existing Package: Example in COVASIM

Although COVID-19 modeling packages are often open source, it may not be desirable to alter their initialization of agents by changing the code to add several factors and dependencies. Indeed, editing the source code of a package (known as ‘forking’) results in creating a new local version for a modeling team, which complicates the possibility of benefiting from updates in future package releases. Consequently, we recommend:

**(R8)** to build on top of existing modeling packages whenever possible, so that future releases (e.g., an optimized COVASIM) can be conveniently used by the team

**Figure 2:**
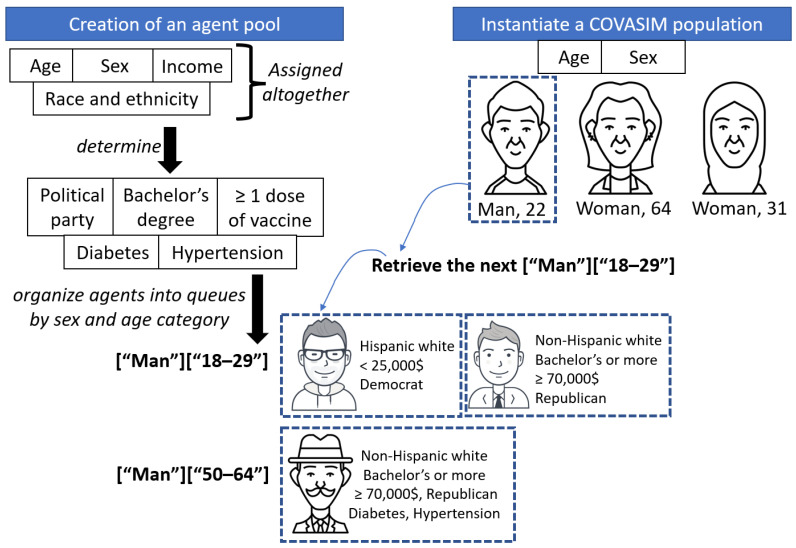
The attributes of COVASIM agents can be expanded by matching them with virtual individuals from a large pool generated from nationally representative U.S. datasets (Table 3). The pool should be larger than the COVASIM population to ensure that each retrieval request for a matching profile can be satisfied. *Icons from User Insights and Domp Icon at icon-icons.com, CC Attribution license (CC BY 4.0)*.

Expanding the characteristics of agents without altering a package’s initialization process can be achieved by matching, illustrated in Figure 2 for COVASIM. First, the modeling package would follow its normal procedure to initialize a set *S* of agents of the desired size. In parallel, modelers would create a larger pool *P* of agents with their required characteristics and dependencies (Section 3.2). Second, we augment the characteristics of each agent in *S* by finding a similar agent (i.e., a ‘match’) in *P*. This requires efficient data structures, as poor implementations could significantly slow down the process of agent creation. For example, an inefficient approach for an agent in *S* would be to scan the entire population *P*, each time computing the distance with an agent in *P*, then finally selecting the most similar one. Once a match in *P* has been found, it must be removed, which should also be done carefully to avoid triggering massive data movements. Research in discrete simulations has often used *hierarchical data structures* (e.g., two or three tiers) to optimize operations [96]. In this case, we need a structure that allows fast lookups to identify a match, hence the attributes offered by the package (e.g., age, sex) can be used as keys in a *dictionary* (Figure 2). We also need a structure that supports quick removals of an individual to move onto the next one, hence a *queue*. 

## 4. Human Errors in Decision-Making

### 4.1. Limitations of Observations and Reflections

There is a broad tendency to create ABMs with highly rational agents [97], which is even more the case in COVID-19 research as models are often grounded in compartmental techniques designed for epidemiology. However, humans are neither mechanical objects nor omniscient, hence individuals *neither* form homogeneous responses to vaccines (e.g., with a set percentage of the entire population taking vaccines) *nor* engage in a comprehensive inventory of all relevant parameters to achieve an optimum (e.g., by maximizing a utility pay-off). Reusing the analogy of Giubilini and Savulescu, refusing vaccination is similar to refusing the use of seat belts when driving [98]: given the balance of extremely rare side effects and the cost of delayed or absent vaccination [99], this decision would not happen if individuals used the evidence-base to maximize their own benefits. Indeed, cognitive sciences show that humans only capture a small portion of the available information [100], sometimes making errors in storing this information, and ultimately interpreting it based on their own heterogeneous beliefs [101]. Prior works in modeling COVID-19 [28,29,33] and perceptual uncertainties in ABMs [102] have already accounted for some of these aspects when creating *cognitive* social simulations [103] that reflect the imperfect, heterogeneous decisions that individuals make on vaccination. While some works introduce the notion of imperfect decisions in COVID-19 by accounting for uncertainty about individual health states [104], research shows that there are at least *three sources of individual errors* to reflect that individuals use social information sub-optimally [105] when observing their peers (with respect to infection, vaccine choices, or death). That is, an agent may only observe some of its peers (*insufficient samples*) and/or pay attention to only some of their information (*superficial observations*) and/or does not sufficiently reflect on the evidence collected (*limited ability*). Consequently, we recommend to:

**(R9)** shift from purely rationale/mechanistic COVID-19 models of human decision-making onto cognitive social simulations that account for imperfection in information capture, its storage, and its use for behavior change.

### 4.2. Operationalizing Human Errors in a Model: The Role of Machine Learning as a Filter

**Figure 3:**
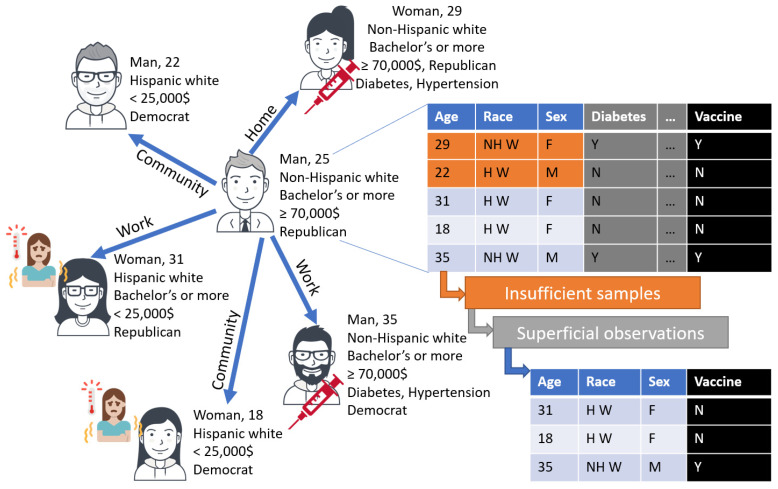
Each agent observes the vaccination decision and attributes of its peers. The initially perfect observations are altered by perceptual limitations and errors, thus producing an observation set with fewer peers and/or fewer attributes. *Icons from User Insights and Coronavirus at icon-icons.com, CC Attribution license (CC BY 4.0)*.

At each simulation tick, each agent can observe the behavior of its peers (e.g., whether to wear a mask, engage in social distancing, wash hands, vaccinate) along with their characteristics. Conceptually, the set of observations can simply be reduced to account for insufficient samples and superficial observations, as shown in Figure 3. However, the main technical difficulty is to ensure that an agent can change based on these observations. The development of such adaptive skills requires a *learning mechanism*. As we recently discussed [106], hybrid agent-based models can deal with adoption behavior in different ways, such as by giving their observations as input to a machine learning model. That is, an agent observes by gathering a data table, reflects by deriving a machine learning model (e.g., why do my friends wear masks?), and acts by applying the model to its own characteristics (e.g., my friends with comorbidities wear a mask and I have comorbidities hence I will wear masks). We thus recommend to:

**(R10)** enable agents to change behavior not only in response to policies (e.g., lockdown) or instincts (e.g., disease avoidance), but also by *learning* from their individual contexts. 

## 5. Discussion

### 5.1. Overview

In the absence of global vaccination, simulation experts have predicted continuous waves of COVID-19 infections [107]. This is now a reality, and most Americans understand that COVID-19 is here to stay [1]. As mutations and natural selection continue to occur, new variants emerge and vaccines need to be continuously redeveloped [14] and adopted by the population [17,18,19,20,21,22]. In the U.S., the federal government has purchased 66 million doses of bivalent boosters that target BA.4 and BA.5 Omicron subvariants [108]. Although the Omicron subvariant BA.5 has been called ‘the worst version’, Bruce Y. Lee explained in a popular piece that “it’s the worst version of what’s been getting *progressively worse*, and you never know when another even worse version will emerge” (emphasis added). [109] Since COVID-19 precautions are going away [15,16], the virus will continue to spread and subvariants will emerge. Expecting the problem to just disappear by believing variants will get gradually weaker may amount to wishful thinking, “like expecting different animal and plant species to get weaker over time.” [108] 

Since Agent-Based Modeling has been the dominating approach for COVID-19 in recent times [110], our scoping review sought to guide the development of future ABMs by examining three dimensions that are essential to effectively pivot into a long-term view. We covered emerging considerations on immunity (e.g., decays, hybrid cases), the growing evidence base on determinants of vaccinal choice and the ability to use them in simulation, and the possibility of shifting towards cognitive social simulations to better capture human decision-making. While recent reviews and recommendations have targeted national health systems and governments [111,112], our ten recommendations are directed at the community on modeling and simulation.

However, ongoing discussions in the scientific community and changes in the evidence base may lead to additional considerations, as discussed below.

### 5.2. Limitations

Our scoping review stressed the importance of psychological and information variables, suggesting that they can be included through a sequential initialization process or by engaging in hybrid modeling (e.g., by using machine learning to create adaptive agents). However, COVID-19 models may ultimately become too complex, given the pursuit to “covering the full behavioral and social complexity of societies under pandemic crisis” [113] through an ever-expanding set of rules and states. The design of COVID-19 models going forward may thus benefit from a shift from a monolithic piece onto a set of integrated sub-models. This shift would come with its own challenges, as risks for model integration have been discussed extensively elsewhere [114]. However, it may be simpler to develop and reuse dedicated modules, since many COVID-19 models have significant overlaps [115]. For example, this would facilitate the integration of a sub-model dedicated to capturing the perceived utility of certain actions (e.g., getting a vaccine) for an agent, such as modeling vaccination as a function of personal beliefs (e.g., via the Theory of Planned Behavior [116]) or through an emphasis on government accountability [117]. 

This paper focused on improving models of COVID-19. However, the actions taken to prevent COVID-19 will also impact other diseases. For instance, facemasks and social distancing can reduce other airborne diseases, while the prioritization of hospital resources for COVID-19 impacts other operations [118]. In addition, there is a growing interest in *coinfection*, particularly for coinfections of COVID-19 and bacteria [119], such as tuberculosis [120]. Consequently, COVID-19 cannot always be designed or expected to be utilized in isolation. Emerging models have started to include COVID-19 alongside another disease [121]. Future COVID-19 models may thus have to be integrated alongside modules for other diseases. Given this requirement, there may be a tradeoff between the sophistication of a COVID-19 model and the ability to integrate it alongside other disease models. 

## Figures and Tables

**Table 1:** Handling of variants and (temporary) immunity in recent COVID-19 models.

		*Handling of Immunity*		
Ref	Variants	Natural	Vaccine	Hybrid
[35]	Delta variant only	Lasts 180 days	Impact on 5 parameters (e.g., death, infection, hospitalization, transmission, asymptomatic) defined via piecewise linear functions. Peak reached 2 weeks after one shot, remains constant for 8 months, then *linear decay* for 6 months. The level of immunity for each linear segment depended on the vaccine used. Booster restores peak vaccination benefit in 1 day.	Exists but unspecified
[36]	Emerge ahead of the winter, every 6 or 12 months	Initial peak at 95%, exponential decay to 20% in 600 days	Initial peak at 85%, *exponential decay* to 15%, half-life 105 days. Higher peak after a booster, but same decay.	None
[57]	Randomly appear 4/6/10 months after past variant	2-part *exponential decay*, with half-life and duration parameters fit to data. Neutralization level depends on variant and vaccine.		None
[58]	Omicron variant only	*Gamma distribution* set either to 9 months (shape 7 and scale 39.11) or one year (shape 3.7 and 98.65).		None
[59]	Omicron variant only	Protection against same variant has exponential duration of mean 1/900, then no protection	After an average of 6 months since the second dose or a booster, individuals transition into a ‘waned vaccine effectiveness’ status.	Exists but unspecified
[55]	Unspecified (2021 data)	Delayed *gamma-distributed* temporary immunity with mean 350 (for vaccines) and 242 days (for recovery)		None
[56]	Delta variant only	Full immunity for entire duration of simulation (March 2020–Nov. 2021)	10% of individuals do not lose immunity, 10% have little protection, the remaining 80% get temporary protection by moving through a series of compartments instead of a single one (which would cause an exponential decay) hence using a *Gamma distribution* with peak efficacy of 92% and decay to 35% over 6 months	None

**Table 2:** Propensity of U.S. individuals in specific socio-demographic categories to get vaccinated or not, and strength of the effect as reported in the references. *We counted a reduction in refusal as being equivalent to an increase in acceptance*.

Determinant	Category	Get Vaccine	Effect Strength	References
Age	18–29	No	Strong	[77,78]
	30–49	Yes		[79]
	50–64	Yes		
	65+	Yes		
Sex	Male	No		[77]
Ethnicity	Non-Hispanic White	Yes		[79]
	Non-Hispanic Black	No		[77,80,81]
	Hispanic	No		
	Asian/Pacific Islander	Yes		[80]
	Other	No		[79,80]
Employment	Unemployed	Yes	Weak	[77]
	Employed (full or part time)	No	Strong	
Education level	Bachelor’s degree or more	Yes		[79,80,82]
	No college degree	No		[80,82]
Annual income	<25,000	No		[81]
	≥70,000	Yes		[80]
Comorbidities	Yes	Yes		
Political party	Republican	No		[79]
	Democrat	Yes		
Prior COVID-19 infection	Yes	Yes		[77]
Know someone who died	Yes	Yes		
Religion	Catholic	No	Weak	

## Data Availability

Not applicable.
